# Persistence and Environmental Dissemination of a Novel *mcr-10.6* Allele in *Enterobacter vonholyi* Across a Poultry Wastewater Treatment System

**DOI:** 10.3390/microorganisms14061182

**Published:** 2026-05-24

**Authors:** Hosana Dau Ferreira de Souza, Thereza Cristina da Costa Vianna, Juliana Ferreira Nunes, Vinícius Carneiro Assunção, Ana Paula Alves do Nascimento, Ramon Loureiro Pimenta, Alexander Machado Cardoso, Maysa Mandetta Clementino, Miliane Moreira Soares de Souza, Irene da Silva Coelho, Kayo Bianco, Shana de Mattos de Oliveira Coelho

**Affiliations:** 1 Department of Veterinary Microbiology and Immunology, Federal Rural University of Rio de Janeiro (UFRRJ), Seropédica, Rio de Janeiro 27323-000, Brazil; hosana.souza@fiocruz.br (H.D.F.d.S.); jufnunes2@gmail.com (J.F.N.); rpimenta27@gmail.com (R.L.P.); milianemss@gmail.com (M.M.S.d.S.); irenecs@yahoo.com (I.d.S.C.); 2 National Institute for Quality Control in Health, Oswaldo Cruz Foundation (FIOCRUZ), Rio de Janeiro 21040-900, Brazil; tpatricio@aluno.fiocruz.br (T.C.d.C.V.); vinicius.assuncao@fiocruz.br (V.C.A.); maysa.mandetta@fiocruz.br (M.M.C.); kayo.bianco@fiocruz.br (K.B.); 3 Department of Biology, Rio de Janeiro State University (UERJ), Rio de Janeiro 23070-200, Brazil

**Keywords:** environmental reservoir, evolutionary hotspot, selection pressure in engineered systems, ARG dissemination pathways

## Abstract

Wastewater treatment plants (WWTPs) are important interfaces for the persistence and dissemination of antimicrobial resistance genes (ARGs) in the environment. This study investigated colistin resistance and the presence of mobile colistin resistance (*mcr*) genes in *Enterobacterales* isolated from a poultry slaughterhouse WWTP in Brazil. Samples were collected from raw sewage, an equalization tank, and treated effluent. A total of 27 *Enterobacter* spp. isolates were identified, of which 70.4% showed resistance to colistin (MIC range: 2 to ≥512 mg/L). PCR screening detected *mcr-1* in two isolates and *mcr-10* in three isolates distributed across all treatment stages, including the final effluent. Whole-genome sequencing of a representative isolate from treated effluent identified *Enterobacter vonholyi* ST3343, carrying a plasmid-borne *mcr-10* gene on an ~107 kb IncFII(Yp) plasmid, along with additional resistance determinants. Phylogenetic analysis supported the classification of this gene as a novel allele, *mcr-10.6*. The persistence of a clonal lineage harboring *mcr-10.6* throughout the treatment system indicates that conventional wastewater treatment may not effectively eliminate clinically relevant ARGs. These findings highlight treated effluent as a potential route for environmental dissemination of colistin resistance and reinforce the need for improved monitoring and mitigation strategies within a One Health framework.

## 1. Introduction

Poultry production is a major component of the global food system, providing an accessible and widely consumed source of animal protein. Brazil is one of the leading producers and exporters of chicken meat, and this sector plays a key role in both food security and the national economy [1]. In intensive production systems, antimicrobials are commonly administered via feed or drinking water for therapeutic, metaphylactic, and prophylactic purposes, particularly to control infections caused by *Enterobacterales*, which are natural inhabitants of the avian gastrointestinal tract. However, the widespread and prolonged use of these compounds exerts strong selective pressure, driving the emergence and dissemination of antimicrobial-resistant bacteria [2,3].

A broad range of antimicrobials, including tetracyclines, fluoroquinolones, β-lactams, aminoglycosides, macrolides, and polymyxins, has been used in poultry production [4]. Among these, colistin has been extensively employed to treat infections caused by Gram-negative bacteria [5]. Its intensive use in food-producing animals has contributed to the silent selection and spread of colistin-resistant populations [6]. Resistance to colistin may arise from chromosomal mutations affecting regulatory systems such as PmrAB and PhoPQ, which alter lipid A and reduce antibiotic binding. In addition, plasmid-mediated resistance, particularly through the mobile colistin resistance (*mcr*) gene family, also relies on lipid A modification, although via a distinct mechanism. Specifically, *mcr* genes encode phosphoethanolamine (pEtN) transferases that catalyze the addition of pEtN to lipid A, reducing the negative charge of the outer membrane and consequently decreasing colistin binding affinity [7,8,9]. This enzymatic mechanism is functionally analogous to chromosomal pathways but is mediated by horizontally transferable elements, significantly enhancing its dissemination potential. Since the first description of *mcr-1* in *Escherichia coli* from food animals in China in 2015, multiple variants (*mcr-2* to *mcr-10*) have been identified across diverse hosts and environmental, animal, and clinical reservoirs worldwide [8,9,10].

Within the *Enterobacteriaceae* family, the genus *Enterobacter* comprises species of both ecological and clinical relevance. Members of the *Enterobacter cloacae* complex (ECC), such as *E. cloacae*, *E. hormaechei*, and *E. bugandensis*, are widely distributed in environmental and host-associated niches [11]. These opportunistic pathogens are increasingly linked to healthcare-associated infections and outbreaks. Their capacity to acquire mobile genetic elements, combined with intrinsic AmpC β-lactamases, contributes to multidrug resistance and poses a growing public health concern [12]. Notably, *E. bugandensis* has been associated with severe neonatal infections [13].

Recent studies have reported the detection of *mcr-9* and *mcr-10* in *Enterobacter* spp. from wastewater, animal, and clinical sources [14]. However, most available data are derived from localized studies, limiting a comprehensive understanding of their environmental dissemination. Agricultural effluents, particularly those associated with intensive animal production, may act as reservoirs of antimicrobial resistance genes (ARGs) and hotspots for horizontal gene transfer [15].

Given that colistin is classified by the World Health Organization as a highest-priority critically important antimicrobial [16], the presence of *mcr*-harboring *Enterobacterales* in agricultural wastewater represents a significant concern within the One Health framework. Investigating the prevalence and genomic context of these resistance determinants in environmental isolates is essential to better understand their dissemination and potential impact on public health [17]. Therefore, this study aimed to investigate the occurrence of colistin resistance and *mcr* genes in *Enterobacterales* isolated from a poultry slaughterhouse wastewater treatment system and to characterize the genomic features of an *Enterobacter vonholyi* strain carrying the novel *mcr-10.6* allele.

## 2. Materials and Methods

### 2.1. Study Setting and Water Sampling

**Figure 1:**
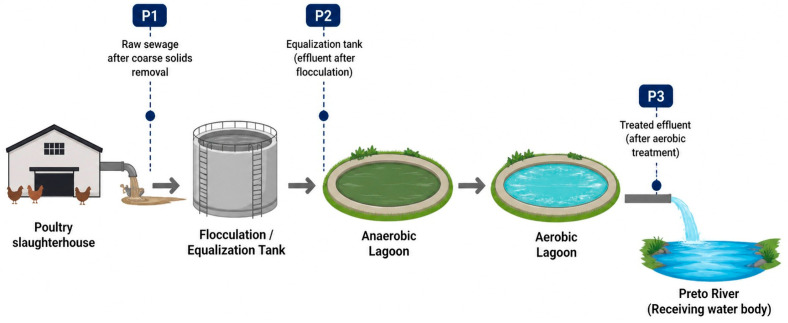
Schematic representation of the poultry slaughterhouse wastewater treatment system and sampling points. Samples were collected at three stages: (P1) raw sewage after coarse solids removal, (P2) equalization/flocculation tank, and (P3) treated effluent after aerobic treatment prior to discharge into the Preto River.

Water samples (500 mL) were collected from a poultry slaughterhouse located in São José do Vale do Rio Preto, Rio de Janeiro State, Brazil (22°12′23.8″ S, 42°57′38.5″ W). Sampling was conducted in June 2022 at three stages of the wastewater treatment system: (i) raw sewage immediately after coarse solids removal (P1), (ii) the equalization tank following pH adjustment and preliminary particle removal (P2), and (iii) treated effluent discharged into the Preto River (P3) (Figure 1). Samples were collected in sterile borosilicate containers, transported at 4 °C, and processed within 6 h of collection.

### 2.2. Bacterial Isolation and Identification

Water samples were concentrated by vacuum filtration through 0.22 μm membranes (Millipore, Burlington, MA, USA). Membranes were transferred to Brain Heart Infusion (BHI) broth and incubated at 36 ± 1 °C for 18–24 h under aerobic conditions. After enrichment, aliquots were streaked onto MacConkey agar and Eosin Methylene Blue (EMB) agar (HiMedia^®^) for selective isolation of Gram-negative bacteria. Colonies with distinct morphologies were subcultured to obtain pure isolates. Bacterial identification was performed using matrix-assisted laser desorption ionization–time of flight mass spectrometry (MALDI-TOF/MS).

### 2.3. Antimicrobial Susceptibility Testing and MIC Determination

Antimicrobial susceptibility testing (AST) was performed using the VITEK^®^ 2 system (BioMérieux, Marcy-L’Etoile, France) according to the manufacturer’s instructions. Thirteen antimicrobials representing different classes were tested: amikacin, amoxicillin/clavulanic acid, cefepime, ceftriaxone, cephalexin, cefuroxime, ciprofloxacin, ertapenem, gentamicin, meropenem, norfloxacin, piperacillin/tazobactam, and trimethoprim/sulfamethoxazole. *Escherichia coli* ATCC 25922 was used as the quality control strain. Colistin minimum inhibitory concentrations (MICs) were determined using the broth microdilution method following BrCAST guidelines [18]. Isolates with MIC values ≥ 2 mg/L were classified as resistant. *E. coli* CCVSU 7107 (*mcr-1* positive control) and *E. coli* ATCC 25922 (negative control) were included.

### 2.4. Detection of Mobile Colistin Resistance (mcr) Genes

The presence of *mcr* genes (*mcr-1* to *mcr-10*) was investigated via PCR using previously described protocols [19,20,21] (Table 1). Genomic DNA was extracted using the Biogene DNA Kit (Bioclin^®^, Belo Horizonte, Brazil) according to the manufacturer’s instructions. PCR reactions (25 μL) contained 0.2 μM of each primer, 1× GoTaq^®^ G2 Master Mix (Promega, Madison, WI, USA), and 20 ng of DNA template. Amplification products were analyzed via electrophoresis on 1.5% agarose gels and visualized under UV light. The following strains were used as positive controls: *E. coli* CCVSU 7107 (*mcr-1*), *E. coli* 12612 (*mcr-2*), *E. coli* CCVSU 9265 (*mcr-3*), *Acinetobacter baumannii* CCVSU 7523 (*mcr-4.3*), *E. coli* CCVSU 11515 (*mcr-5*), and *E. coli* CCVSU 12181 (*mcr-9*).

### 2.5. Multilocus Sequence Typing

Multilocus sequence typing (MLST) was performed according to the scheme described by Miyoshi-Akiyama et al., 2013 [22], targeting seven housekeeping genes (*dnaA*, *fusA*, *gyrB*, *leuS*, *pyrG*, *rplB*, and *rpoB*). PCR products were sequenced using the BigDye™ Terminator v3.1 Cycle Sequencing Kit (Thermo Fisher Scientific, Waltham, MA, USA) and analyzed on a SeqStudio Genetic Analyzer (Thermo Fisher Scientific). Sequence quality was assessed using Sequencing Analysis Software v7.0, and bases with quality scores below Q30 were trimmed. Alleles and sequence types (STs) were assigned using the PubMLST database for *Enterobacter* spp. MLST was performed as an initial screening approach to assess the clonal relatedness among *mcr*-positive isolates recovered from different stages of the wastewater treatment system. This strategy enabled the identification of potentially persistent lineages across the system prior to the selection of a representative isolate for whole-genome sequencing. Subsequent WGS analysis was used to confirm and further characterize the identified sequence type at higher resolution.

### 2.6. Whole-Genome Sequencing and Bioinformatic Analyses

Only one isolate carrying *mcr-10* and recovered from treated effluent (P3) was selected for whole-genome sequencing. Whole-genome sequencing was performed using short-read Illumina technology, and no long-read sequencing approach was applied. Genomic libraries were prepared using the Illumina DNA Prep Kit (Illumina, San Diego, CA, USA) and sequenced on the Illumina MiSeq platform to generate paired-end reads. Raw reads were quality-checked using FastQC v0.12.1 and summarized with MultiQC v1.35. PhiX174 bacteriophage (PhiX), a small single-stranded DNA virus commonly used as an internal control in Illumina sequencing runs, sequences were removed using BBDuk (v39.15), and reads were processed with Fastp v1.3.3 (Phred score ≥ 20) for adapter trimming and quality filtering [23]. *De novo* assembly was performed using Unicycler v0.5.1 [24], followed by polishing with PolyPolish v0.6.1 [25] based on alignments generated with Burrows–Wheeler Aligner using the Maximal Exact Matches algorithm (BWA-MEM, v2.3) [26]. Genome completeness was assessed using BUSCO v5.8 [27], and contamination was evaluated using GUNC v1.1.1 [28]. Plasmid sequences were identified using MOB-recon (MOB-suite, v3.1.9) and typed with MOB-typer (MOB-suite, v3.1.9) [29]. Chromosomal assemblies were additionally screened with geNomad v1.12.0 [30] to detect plasmid-like elements. Taxonomic verification of the assembled genome was performed using the Type (Strain) Genome Server (TYGS) database [31]. This platform employs genome-based taxonomic analysis, including digital DNA–DNA hybridization (dDDH) estimation and phylogenomic comparison against reference type strain genomes, allowing accurate species-level classification. Antimicrobial resistance genes were identified using Resistance Gene Identifier (RGI, v6.0.8) with the CARD database [32] and AMRFinderPlus v4.2.7 [33]. Assemblies were also screened with ABRicate against multiple databases for resistance, virulence, plasmid, and metal resistance markers. The pathogenic potential of the isolate was predicted using PathogenFinder v2.0 [34].

### 2.7. Evaluation of the mcr-10.6 Allelic Variant

Reference sequences of *mcr-10* alleles were retrieved from the NDARO/NCBI Reference Gene Catalog (database version: 2026-01-21.1). Sequences were aligned and translated into amino acid sequences using CLC Genomics Workbench v26 (Fiocruz). Phylogenetic analyses were performed based on nucleotide and deduced amino acid complete sequences of mcr-10 alleles, rather than whole-genome sequences, using the Maximum Likelihood method with 1000 bootstrap replicates. The newly identified allele was submitted to NDARO and assigned the designation *mcr-10.6*.

## 3. Results

### 3.1. Isolation and Colistin Resistance

A total of 27 *Enterobacter* spp. isolates were recovered from wastewater samples collected along the slaughterhouse treatment system. Most isolates were assigned to the *Enterobacter cloacae* complex (ECC) based on MALDI-TOF/MS identification. Among them, 19 isolates (70.4%) exhibited resistance to colistin, as determined via broth microdilution, with minimum inhibitory concentrations (MICs) ranging from 2 to >512 μg/mL. Resistant isolates were distributed across all sampling points, including raw sewage (P1; *n* = 9), the equalization tank (P2; *n* = 6), and treated effluent (P3; *n* = 4).

### 3.2. Detection of mcr Genes

Among the 19 colistin-resistant isolates, two carried the *mcr-1* gene (P1 and P2), while three harbored *mcr*-10 (P1, P2, and P3). No other *mcr* variants (*mcr-2* to *mcr-9)* were detected. Antimicrobial susceptibility profiles of these isolates are presented in Table 2.

### 3.3. Clonal Relatedness

All *mcr-10*-harboring isolates were assigned to the same sequence type, ST3343, based on MLST screening, indicating clonal relatedness among isolates from different treatment stages. This finding guided the selection of a representative isolate for whole-genome sequencing, which subsequently confirmed the lineage and enabled deeper genomic characterization.

### 3.4. Genomic Features of the mcr-10-Harboring Strain

A representative isolate recovered from treated effluent (P3) was selected for whole-genome sequencing and identified as *Enterobacter vonholyi*. Genome assembly resulted in a high-quality draft genome with 98.6% completeness (Table 3).

PathogenFinder predicted a >96% probability of human pathogenicity. Resistome analysis identified multiple resistance determinants, including *blaACT-91* (AmpC β-lactamase), *fosA* (fosfomycin resistance), and *oqxAB*, associated with efflux-mediated resistance to quinolones and phenicols. Resistome analysis revealed the presence of multiple antimicrobial resistance genes associated with different classes, including β-lactams (*blaACT-91*), fosfomycin (*fosA*), quinolones and phenicols (*oqxAB*), and polymyxins (*mcr-10.6*). Genome annotation using PGAP additionally identified *ampE*, a gene involved in the regulation of AmpC β-lactamase expression. However, *ampE* does not encode a β-lactamase enzyme and does not directly confer resistance. Instead, it acts as a regulatory component of the AmpC expression system, potentially influencing β-lactam susceptibility under specific conditions [35,36,37]. Therefore, only *bla_ACT-91_* was considered a β-lactamase antimicrobial resistance gene (ARG) in this study. A complete resistome profile is presented in Table 4.

Additionally, genome annotation identified a protein annotated as CrrC (MHC0025732.1), which has been associated with regulatory pathways involved in colistin resistance. However, CrrC is not considered a direct antimicrobial resistance gene (ARG), as it does not directly confer resistance. Instead, it acts as a regulatory component of two-component systems (CrrAB/PmrAB) that may modulate lipid A modification pathways. In the absence of known activating mutations or functional evidence, its presence alone is insufficient to infer a resistance phenotype. Therefore, this element was not included in the ARG profile reported in this study.

**Figure 2:**
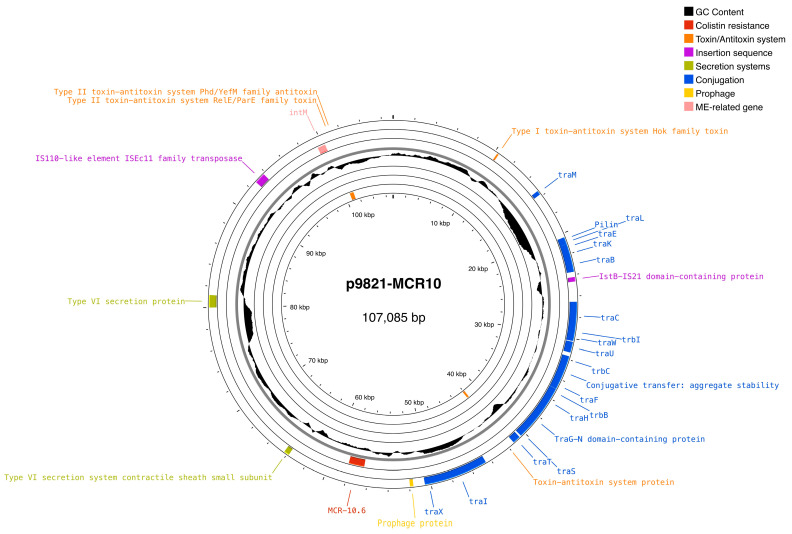
Schematic representation of the IncFII(Yp) plasmid identified in *E. vonholyi* strain CCVSU9821. Protein-coding genes on the forward and reverse strands are shown as rectangles on the outer and inner rings, respectively. Functional categories, including colistin resistance, toxin–antitoxin systems, insertion sequences, secretion systems, conjugation-related genes, prophage regions, and mobile element-related genes, are indicated by distinct colors. The innermost circle represents GC-skew, while the adjacent inner circle shows GC content (deviation from the average) in black.

A single plasmid, assembled as a single contig, was identified in the sequenced strain, corresponding to an approximately 107 kb IncFII(Yp) replicon harboring the *mcr*-*10.6* gene along with additional antimicrobial resistance determinants (Figure 2). Virulence-associated loci related to stress response, adhesion, and iron acquisition were also identified.

#### Description of the Novel Allele *mcr-10.6*

**Figure 3:**
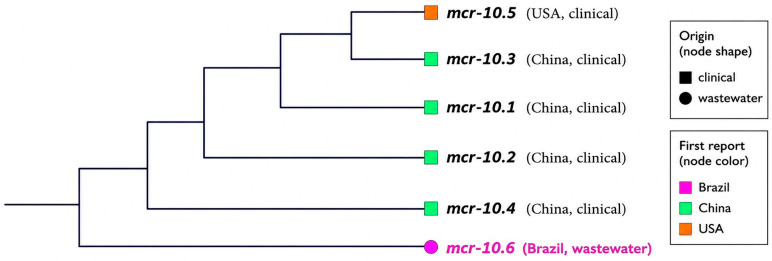
Phylogenetic reconstruction of mcr-10 alleles. The *mcr-10.6* allele identified in this study is highlighted in pink. Colors indicate the country of first report, and symbol shapes represent the source of isolation. Phylogenetic analyses based on nucleotide and amino acid sequences yielded identical topologies; therefore, only one representative tree is shown for clarity.

Phylogenetic analyses based on nucleotide and amino acid sequences of *mcr* alleles (*mcr-1* to *mcr-12*) revealed well-defined clustering within the *mcr* gene family. The sequence obtained from strain CCVSU9821 consistently clustered within the *mcr-10* clade, closely related to previously described *mcr-10* variants. However, it formed a distinct branch within this group, supporting its classification as a novel allele. This topology was consistently observed in both nucleotide and amino acid phylogenies, clearly separating the sequence from other *mcr* families (*mcr-1* to *mcr-4*) (Figure 3). Based on these findings, the allele was designated *mcr*-10.6, as assigned by the NDARO/NCBI Reference Gene Catalog.

## 4. Discussion

The emergence and environmental dissemination of plasmid-mediated colistin resistance represent a major concern for global public health, particularly considering the critical importance of colistin as a last-resort antimicrobial for the treatment of infections caused by multidrug-resistant Gram-negative bacteria [7,16]. In the present study, we identified a novel *mcr-10* allele, designated *mcr-10.6*, in *E. vonholyi* isolates recovered from different stages of a poultry slaughterhouse wastewater treatment plant in Brazil. To the best of our knowledge, this is the first report describing the persistence of the novel *mcr-10.6* allele across an entire poultry wastewater treatment system, including its detection in treated effluent destined for environmental discharge.

Phylogenetic analyses based on both nucleotide and amino acid sequences demonstrated that the detected sequence clustered consistently within the *mcr-10* family while forming a distinct branch relative to previously described variants, supporting its classification as a novel allele. The identification of *mcr-10.6* expands the known diversity of mobile colistin resistance determinants and reinforces the dynamic evolutionary processes driving the diversification of the *mcr* family. Since the initial description of *mcr-1* in 2015, several mobile colistin resistance genes and allelic variants have been reported worldwide, reflecting the remarkable capacity of *Enterobacterales* to acquire and disseminate resistance determinants through horizontal gene transfer [9]. Among these variants, *mcr-9* and *mcr-10* have increasingly attracted attention due to their broad host range, association with mobile genetic elements, and occurrence in environmental, animal, and clinical settings [8,10,14,38].

Although *mcr-10* has been previously reported in *Enterobacterales* from clinical, animal, and environmental sources, available studies remain limited compared with those involving *mcr-1* [10,14]. Recent reports from Asia, Europe, and South America suggest that *mcr-10* is becoming more widely distributed across distinct ecological niches [10,14]. Mentasti et al. (2021) identified ten distinct mobile colistin resistance variants (*mcr-1* to *mcr-10*), reinforcing the growing diversity of plasmid-mediated colistin resistance determinants [20]. Recent studies have also described the emergence of *mcr-11.1* in clinical *Leclercia adecarboxylata* isolates from China and the identification of *mcr-12* in environmental bacteria recovered from metal-contaminated sediments, demonstrating the continuous evolution and diversification of the *mcr* family [39,40]. In Brazil, environmental studies have already demonstrated the occurrence of *mcr-10.1* in surface waters associated with cephalosporin-resistant *Enterobacterales* [41]. However, the detection of the novel *mcr-10.6* allele in a poultry slaughterhouse WWTP highlights the role of animal production-associated wastewater systems as potential reservoirs and dissemination routes for emerging antimicrobial resistance genes.

An important finding of this study was the localization of *mcr-10.6* on an approximately 107 kb IncFII(Yp) plasmid. IncF plasmids are recognized for their adaptability, stability, and ability to mediate the dissemination of antimicrobial resistance determinants among *Enterobacterales* [10,42]. Previous investigations have demonstrated that IncF-type plasmids frequently carry clinically relevant resistance genes, including carbapenemases, extended-spectrum β-lactamases, and *mcr* variants [10,42]. The identification of *mcr-10.6* within this plasmid background reinforces concerns regarding its dissemination potential through horizontal gene transfer.

Additionally, the genomic context surrounding *mcr-10.6* included insertion sequences and mobile element-associated genes, including transposase-related elements from the ISEC11 and IS21 families. Mobile genetic elements play a fundamental role in the mobilization, recombination, and persistence of antimicrobial resistance genes, facilitating their transfer between plasmids, chromosomes, and bacterial hosts [42]. The coexistence of *mcr-10.6* with these elements may contribute to its evolutionary diversification and environmental persistence. The sequenced *E. vonholyi* ST3343 isolate also exhibited a multidrug resistance profile, harboring additional resistance determinants, including *_blaACT-91_*, *fosA*, and *oqxAB* [35]. The high prevalence of AmpC β-lactamases among members of the Enterobacter cloacae complex has been extensively documented, with *bla_ACT_* variants representing some of the most frequently identified resistance determinants in *Enterobacter* spp. [35]. Phenotypic resistance to multiple β-lactams and colistin was consistent with the resistome profile identified via whole-genome sequencing. It is important to distinguish between structural β-lactamase genes and regulatory elements. In this study, *ampE* was identified via genome annotation but was not classified as an ARG, as it is involved in the regulation of AmpC expression rather than encoding a β-lactamase enzyme. This distinction is essential to avoid overestimation of the resistome [35,36,37].

The presence of *bla_ACT-91_* likely contributed to the observed reduced susceptibility to cephalosporins and other β-lactams, while *oqxAB* may be associated with decreased susceptibility to quinolones and phenicols [35,36,37]. Phenotypic resistance to multiple β-lactams observed in this study is consistent with previous reports linking AmpC production to resistance against third-generation cephalosporins and its clinical relevance in bloodstream infections caused by *Enterobacterales* [36,37]. The coexistence of multiple resistance determinants within the same strain raises additional concerns regarding co-selection processes in environments exposed to antimicrobial residues and other selective pressures. It is important to note that regulatory elements such as CrrC were identified in the genome but were not classified as ARGs, as they do not directly confer resistance. Instead, their contribution to colistin resistance depends on specific mutations and activation of regulatory pathways involved in lipid A modification [7,35,36].

Another relevant observation was the persistence of a single clonal lineage, *E. vonholyi* ST3343, throughout the wastewater treatment process, including in the treated effluent. Similar patterns have been reported in hospital and municipal wastewater systems, where antimicrobial resistance genes persist even after conventional or advanced treatment processes [43]. This finding suggests that conventional treatment processes employed by the studied poultry slaughterhouse were insufficient to completely eliminate antimicrobial-resistant bacteria and clinically relevant resistance determinants. Wastewater treatment plants have increasingly been recognized as ecological interfaces where antimicrobial residues, resistant bacteria, and mobile genetic elements coexist under conditions favorable for selection and genetic exchange [15,17,43].

The detection of *mcr-10.6*-positive isolates in treated effluent is particularly concerning because it indicates the potential release of resistant bacteria and plasmid-mediated resistance genes into aquatic environments. Once introduced into natural ecosystems, these determinants may spread to environmental microbiota, wildlife, livestock, and potentially human-associated bacterial populations. This scenario reinforces the importance of adopting a One Health perspective to address antimicrobial resistance, integrating environmental, animal, and human health surveillance. Our findings demonstrate that poultry slaughterhouse wastewater systems may act not only as reservoirs but also as evolutionary hotspots for emerging antimicrobial resistance determinants. The identification of a novel *mcr* allele persisting throughout the treatment process highlights the urgent need for enhanced environmental surveillance, genomic monitoring, and implementation of more efficient wastewater treatment strategies capable of reducing the environmental dissemination of antimicrobial resistance genes.

## 5. Conclusions

This study demonstrated the occurrence and persistence of colistin-resistant *Enterobacter* spp. throughout different stages of a poultry slaughterhouse wastewater treatment system in Brazil. Among the 19 colistin-resistant isolates recovered, *mcr-1* and *mcr-10* were detected, including the identification of a novel *mcr-10 allele*, designated *mcr-10.6*. Whole-genome sequencing revealed that the *mcr-10.6* allele was carried by *E. vonholyi* ST3343 and located on an approximately 107 kb IncFII(Yp) plasmid associated with additional antimicrobial resistance determinants, including *bla_ACT-91_*, *fosA*, and *oqxAB*. Phylogenetic analyses supported the classification of this sequence as a distinct *mcr-10* allelic variant, expanding the currently known diversity of plasmid-mediated colistin resistance genes. The persistence of the same *E. vonholyi* ST3343 clonal lineage across all treatment stages, including in the final treated effluent, indicates that the conventional wastewater treatment process evaluated in this study was insufficient to completely eliminate clinically relevant antimicrobial-resistant bacteria and resistance genes. The detection of plasmid-mediated colistin resistance determinants in treated effluent destined for environmental discharge highlights the potential role of poultry wastewater systems as reservoirs and dissemination routes for emerging antimicrobial resistance genes. These findings reinforce the importance of continuous environmental surveillance, genomic monitoring of wastewater-associated bacteria, and the implementation of more effective wastewater treatment technologies to reduce the environmental spread of antimicrobial resistance. From a One Health perspective, our results strengthen the evidence linking intensive animal production, environmental contamination, and the dissemination of clinically relevant resistance determinants. Coordinated actions integrating animal, environmental, and public health sectors will be essential to preserve the effectiveness of critically important antimicrobials such as colistin.

## Figures and Tables

**Table 1:** Specific primers for detection of *mcr* genes.

Target Gene	Primer Sequence (5′-3′)	Fragment Size (bp)	Reference
*mcr-1*	F: AGTCCGTTTGTTCTTGTGGC	320	Rebelo et al., 2018 [21]
	R: AGATCCTTGGTCTCGGCTTG		
*mcr-2*	F: CAAGTGTGTTGGTCGCAGTT	715	
	R: TCTAGCCCGACAAGCATACC		
*mcr-3*	F: AAATAAAAATTGTTCCGCTTATG	929	
	R: AATGGAGATCCCCGTTTTT		
*mcr-4*	F: TCACTTTCATCACTGCGTTG	1116	
	R: TTGGTCCATGACTACCAATG		
*mcr-5*	F: ATGCGGTTGTCTGCATTTATC	1644	
	R: TCATTGTGGTTGTCCTTTTCTG		
*mcr-6*	F: AGCTATGTCAATCCCGTGAT	252	Borowiak et al., 2020 [19]
	R: ATTGGCTAGGTTGTCAATC		
*mcr-7*	F: GCCCTTCTTTTCGTTGTT	551	
	R: GGTTGGTCTCTTTCTCTCGT		
*mcr-8*	F: TCAACAATTCTACAAAGCGTG	856	
	R: AATGCTGCGCGAATGAAG		
*mcr-9*	F: TTCCCTTTGTTCTGGTTG	1011	
	R: GCAGGTAATAAGTCGGTC		
*mcr-10*	F: GCAATAACCCGACGCTGAAC	366	Mentasti et al., 2021 [20]
	R: GTAACGCGCCTTGCATCATC		

**Table 2:** The table presents the tested antimicrobials and their respective MICs in *mcr-10*-positive isolates at all treatment stages.

	Strains	9844	9992	9821
	Stage	P1	P2	P3
Class of Antimicrobial	Antimicrobial	MIC (mg/L)		
Beta-lactam	Amoxicillin/clavulanic acid	≥32	≥32	≥32
	Piperacillin/Tazobactam	≥128	≥128	≥128
	Cephalexin	≥64	≥64	≥64
	Cefuroxime	≥64	≥64	≥64
	Ceftriaxone	1	32	32
	Cefepime	0.5	2	2
	Ertapenem	1	4	≥8
	Meropenem	1	2	2
Aminoglycoside	Amikacin	2	2	2
	Gentamicin	≤1	≤1	≤1
Fluoroquinolone	Ciprofloxacin	≤0.06	≤0.06	≤0.06
	Norfloxacin	≤0.5	≤0.5	1
Diaminopyrimidine/sulfonamide	Trimethoprim/sulfamethoxazole	≤20	≤20	≤20
Polymyxin	Colistin	≥512	≥512	≥512

**Table 3:** Key genomic features of *E.* CCVSU9821.

Strain	CCVSU9821
Size	4,736,078
GC content (%)	55.4
N50	345,175
Number of Contigs (with PEGs)	66
Number of coding sequences	4703
Number of RNAs	75

**Table 4:** ARGs detected in the *E. vonholyi* CCVSU9821 genome.

Resistance Gene(s)	Identity (%)	Coverage (%)	Genomic Location	Mechanism	Antimicrobial Class	Antimicrobial
*mcr-10.6*	100	100	Plasmid; IncFII(Yp)	Lipid A modification	Polymyxins	Colistin
*bla_ACT-91_*	98.95	100	Chromosome	AmpC β-lactamase	β-lactams	Amoxicillin/clavulanate, Ceftriaxone and Cefepime
*oqxA*, *oqxB*	92.33/99.52	100	Chromosome	Efflux pump	Quinolones	Ciprofloxacin, Norfloxacin
*fosA*	98.83	100	Chromosome	Fosfomycin-modifying enzyme	Fosfomycin	Fosfomycin

## Data Availability

The draft genome assembly has been deposited in GenBank under the accession numbers JBSSTC010000001–JBSSTC010000066. The associated BioProject accession is PRJNA1373205.

## Abbreviations

The following abbreviations are used in this manuscript:

WWTPs	Wastewater Treatment Plants
ARGs	Antimicrobial Resistance Genes
MCR	Mobile Colistin Resistance
ECC	*Enterobacter cloacae* Complex
BHI	Brain Heart Infusion
EMB	Eosin Methylene Blue
MALDI-TOF/MS	Matrix-Assisted Laser Desorption Ionization–Time of Flight Mass Spectrometry
AST	Antimicrobial Susceptibility Testing
STs	Sequence Types
